# A Nonstructural Protein Responsible for Viral Spread of a Novel Insect Reovirus Provides a Safe Channel for Biparental Virus Transmission to Progeny

**DOI:** 10.1128/JVI.00702-19

**Published:** 2019-07-17

**Authors:** Qian Chen, Kris Godfrey, Jiejie Liu, Qianzhuo Mao, Yen-Wen Kuo, Bryce W. Falk

**Affiliations:** a Department of Plant Pathology, University of California, Davis, California, USA; b Contained Research Facility, University of California, Davis, California, USA; c Fujian Province Key Laboratory of Plant Virology, Institute of Plant Virology, Fujian Agriculture and Forestry University, Fuzhou, Fujian, People’s Republic of China; Instituto de Biotecnologia/UNAM

**Keywords:** *Diaphorina citri*, *Diaphorina citri* reovirus, biparental transmission, insect reovirus, sperm, transovarial transmission, tubule

## Abstract

*Diaphorina citri reovirus* (DcRV) was previously identified based on metagenomics surveys for virus discovery. Here, we demonstrated that DcRV induces persistent infection in its psyllid host, *Diaphorina citri*. DcRV was efficiently vertically passed to offspring in a biparental manner. Transmission electron microscopic and immunological analyses showed that the DcRV-encoded nonstructural protein P10 assembled into a virion-packaging tubular structure which is associated with the spread of DcRV throughout the bodies of *D. citri* insects. P10 tubules containing virions were associated with oocytes of female and sperm of male *D. citri* insects, suggesting a role in the highly efficient biparental transmission of DcRV. Knocking down P10 by RNA interference for males reduced the percentage of DcRV-infected progeny and for females reduced the viral accumulation in progeny. These results, for the first time, show that a nonstructural protein of a novel insect reovirus provides a safe and pivotal channel for virus spread and biparental transmission to progeny.

**IMPORTANCE** The Asian citrus psyllid, Diaphorina citri Kuwayama, is an important pest in the worldwide citrus industry. It is the vector of “*Candidatus* Liberibacter asiaticus,” the bacterial pathogen of Huanglongbing, which is currently considered the most destructive disease of citrus worldwide. DcRV was previously identified based on metagenomics surveys for virus discovery. Here, we found that this novel and persistent insect reovirus took advantage of a virus-encoded nonstructural protein, P10, for efficient vertical transmission from parents to progeny. P10 assembled into a virion-packaging tubular structure and was associated with oocytes of female *D. citri* and sperm of males. Consistent with this, knockdown of P10 for either male or female *D. citri* insects inhibited DcRV transmission to offspring. This tubular strategy for viral spread and biparental transmission might serve as a target for controlling viral vertical transmission and population expansion.

## Abstract (precis)

The Asian citrus psyllid, Diaphorina citri Kuwayama, is an important pest in the worldwide citrus industry. It is the vector of “*Candidatus* Liberibacter asiaticus,” the bacterial pathogen of Huanglongbing, which is currently considered the most destructive disease of citrus worldwide. DcRV was previously identified based on metagenomics surveys for virus discovery. Here, we found that this novel and persistent insect reovirus took advantage of a virus-encoded nonstructural protein, P10, for efficient vertical transmission from parents to progeny. P10 assembled into a virion-packaging tubular structure and was associated with oocytes of female *D. citri* and sperm of males. Consistent with this, knockdown of P10 for either male or female *D. citri* insects inhibited DcRV transmission to offspring. This tubular strategy for viral spread and biparental transmission might serve as a target for controlling viral vertical transmission and population expansion.

## INTRODUCTION

The Asian citrus psyllid, Diaphorina citri Kuwayama (Hemiptera: Liviidae), is an important pest in the worldwide citrus industry. It is the vector of “*Candidatus* Liberibacter asiaticus” (CLas), the bacterial pathogen of Huanglongbing (HLB), which is currently considered the most destructive disease of citrus worldwide (1). Previous surveys of the *D. citri* population in Florida for insect viruses identified two RNA fragments for which sequence analysis suggested phylogenetic similarity with *Nilaparvata lugens reovirus* (NLRV) of the genus *Fijivirus* in the family *Reoviridae* (2). These authors suggested this is a putative reovirus called *Diaphorina citri reovirus* (DcRV) (2). Subsequent metagenomics surveys of *D. citri* populations using small RNA and transcriptome sequencing by our laboratory showed that *D. citri* populations from Florida, Hawaii, and China contained a virus homologous to DcRV (3). Seven partial RNA sequences (S1, S2, S3, S4, S7, S8, and S10), ranging from 1,216 to 4,454 nucleotides (nt) in length, displayed nucleotide homology and deduced amino acid sequence similarity with NLRV. However, whether DcRV is a real virus or if these sequences could be representative of endogenous viral elements was not known (4).

Reoviruses (family *Reoviridae*) have nonenveloped and icosahedral virions (5). The virions are two- or three-layered icosahedra, approximately 60 to 80 nm in diameter (5). Viral genomes consist of 9 to 12 segments of linear double-stranded RNA (dsRNA) (S1 to S12), encoding structural proteins and nonstructural proteins (5). Reoviruses are very widespread, with hosts including vertebrates, invertebrates, plants, and fungi. NLRV of the genus *Fijivirus* has been shown so far to infect and replicate only in Nilaparvata lugens, while other members of this genus infect both plants and their insect vectors. So far, studies on NLRV have focused on the genome sequences and the means of transmission (6–8), but other information, including the functions of viral proteins, molecular variation, and pathogenicity for insect hosts, is not known. Despite well-documented studies describing the morphology or genomes of other reo-like viruses in insects (9–11), further research is required. Therefore, the understanding of insect reoviruses, including the function of viral proteins, biological characteristics of the viruses, viral infection, and transmission mechanisms, as well as host range, have lagged behind those of vertebrate- and plant-infecting reoviruses. Among the proteins encoded by plant-infecting reoviruses, a virus-encoded nonstructural transmembrane protein has been shown to assemble into tubular structures and package virions for viral intercellular spread within the insect vector (12–14). However, for insect-specific reoviruses, we do not know whether proteins generating similar tubular structures are also encoded by these viruses and, if so, whether they play similar functions in their insect hosts.

In the present study, we confirmed that DcRV infects and replicates within *D. citri*. By combining immunofluorescence and transmission electron microscopy, together with microinjection-based RNA interference (RNAi), we also showed that the DcRV-encoded nonstructural protein P10 is associated with tubules which package DcRV virions for spread within *D. citri*. More importantly, this tubule strategy used by DcRV provided a safe channel to facilitate the DcRV biparental transmission to progeny.

## RESULTS

### DcRV widely distributes in the body of *D. citri* insects.

**FIG 1:**
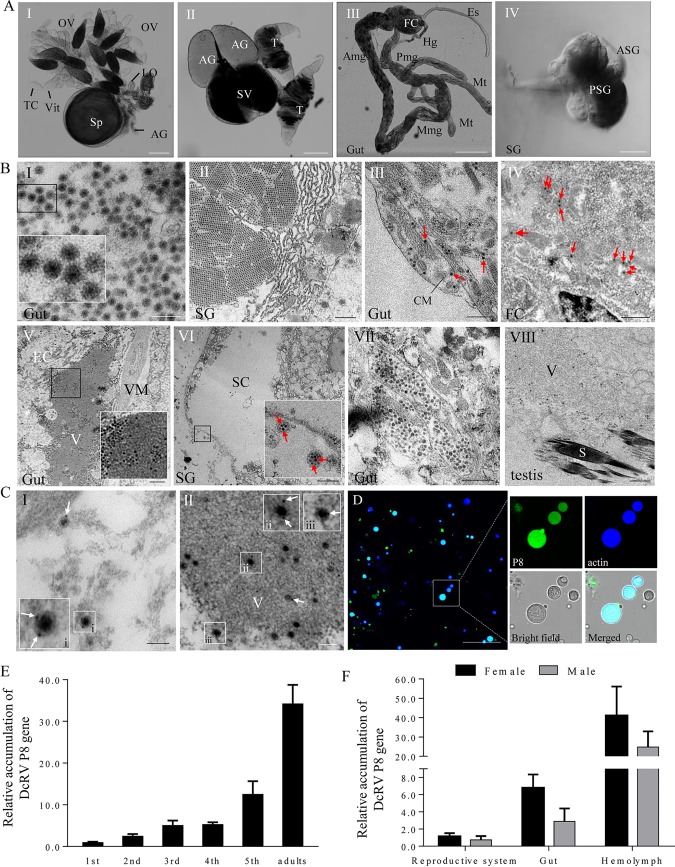
DcRV particles are widely distributed throughout the organs of *D. citri*. (A) Transmitted light micrograph of dissected organs of *D. citri*. The organs mainly consist of the reproductive system (I and II), gut (III), and salivary gland (IV). Bars are 200 μm (I, II, and III) and 50 μm (IV). (B) Electron micrographs showing DcRV virions aggregated into crystalline arrays, scattered throughout the cytoplasm, distributed at the periphery viroplasm, or contained in vesicular compartments in different *D. citri* organs. Insets are enlarged images of the boxed areas in each panel. Arrows indicate the free virions in the tissues. Bars are 200 nm (I), 700 nm (II, VII, and VIII), 500 nm (III and IV), 1 μm (V), and 3 μm (27). OV, ovary; LO, lateral oviduct; Sp, spermatheca; AG, accessory gland; TC, trophic chamber; Vit, vitellarium; T, testis; SV, seminal vesicle; Es, esophagus; FC, filter chamber; Amg, anterior midgut; Mmg, middle midgut; Pmg, posterior midgut; Hg, hind gut; Mt, Malpighian tubule; SG, salivary gland; PSG, principle salivary gland; ASG, accessory salivary gland; CM, circular muscle; EC, epithelial cell; V, viroplasm; VM, visceral muscle; SC, salivary cavity; S, sperm. (C) Immunogold labeling of P8 on outer shells of virions in the cytoplasm (I) and on the viroplasm (II) of epithelial cells of guts. White arrows mark gold particles. Insets i, ii, and iii are enlarged images of the boxed areas of panels I and II. Bars, 100 nm. (D) Immunofluorescence microscopy showing that DcRV is distributed in the hemolymph of DcRV-infected *D. citri*. Hemocytes were immunolabeled with P8-FITC antibody (green) and actin dye phalloidin-Alexa Fluor 647 carboxylic acid (blue) and presented single sections examined by confocal microscopy. Bars, 50 μm. (E) RT-qPCR showing that DcRV accumulation gradually increased with the development of *D. citri*. (F) RT-qPCR showing gender preference and tissue-specific distribution of DcRV. The results were normalized against the level of the actin mRNA, and the gene accumulation levels of P8 in 1st-instar nymphs (E) or in reproductive systems of males (F) was normalized as 1. Means (±standard deviations [SD]) from 3 biological replicates are shown.

Small RNA and transcriptome sequencing coupled with reverse transcription-PCR (RT-PCR) detection showed that *D. citri* from Hawaii was infected by only DcRV (3). In attempts to identify DcRV virions, the organs of *D. citri* were dissected and prepared for examination by transmission electron microscopy (TEM) (Fig. 1A). TEM examination of thin sections showed abundant virus­like particles typical of reoviruses (Fig. 1B). The particles appeared spherical, approximately 70 nm in diameter, and possessed a double-layered shell (Fig. 1BI). They were composed of an electron-dense central core and a capsid of moderate electron density, which corresponded to the morphology of reovirus virions. These were distributed throughout all tissues and organs, including the gut, fat bodies, salivary glands, and female and male reproductive systems (Fig. 1B). They were often aggregated into crystalline arrays of various sizes, sometimes scattered in a free form throughout the cytoplasm, distributed at the periphery or inside the electron-dense viroplasms (Fig. 1B). Some particles were also found in vesicular compartments in the vicinity of the cell membrane (Fig. 1BVI). About 20 *D. citri* insects were examined to better understand the distribution and form of virions in different organs (Table 1). Immunoelectron microscopy showed that specific IgG of P8, the predicted major outer capsid protein encoded by the S8 genome segment of DcRV, specifically recognized these particles, such as the free virions in the cytoplasm and the intact particles within the viroplasm (Fig. 1C). The P8-specific IgG also reacted speciﬁcally with the matrix of the viroplasm, which was consistent with the fact that viral proteins were produced in the viroplasm. The immunofluorescence microscopy showed that P8-specific IgG also bound to the hemocytes, indicating that DcRV was present in the hemolymph (Fig. 1D). The uninfected California *D. citri* insects were DcRV negative in each organ in the TEM examination and in the hemolymph of immunofluorescence microscopy (data not shown).

**TABLE 1:** Distribution and form of virions in different organs

Form of virions	Organs^*a*^				
	Filter chamber	Gut	Salivary gland	Ovary	Testis
Crystalline arrays	+	+	+	+	+
Free virions	+	+	+	+	+
At the periphery or inside the viroplasms	+	+	+	+	+
In vesicular compartments	−	+	+	+	−

a +, Positive; −, unobserved.

We next performed developmental expression analysis to assess DcRV accumulation in nymphs and adults of *D. citri*. The accumulation of the S8 genome segment was used for DcRV accumulation. DcRV S8 accumulation gradually increased with insect development from the 1st-instar nymph to adult and peaked in adults (Fig. 1E). Gender- and tissue-specific accumulation of DcRV showed the overall higher accumulation of DcRV in female adults than in male adults. The highest accumulation was found in the hemolymph for adults of both genders, indicating that hemolymph was more compatible for virus accumulation (Fig. 1F).

### DcRV is transmitted in a vertical manner.

**FIG 2:**
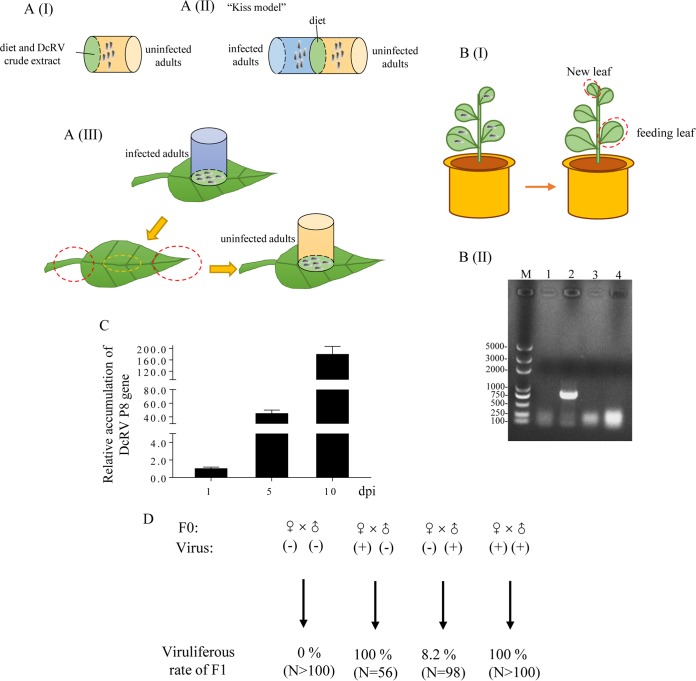
DcRV can be vertically transmitted by *D. citri*. (A) Schematic illustration of the feeding systems. (I) Conventional membrane feeding with diet mixed with DcRV crude extracts. (II) Kiss model for DcRV delivery and acquisition via the liquid diet. DcRV-infected and uninfected *D. citri* adults were confined in two individual cages, between which the diet was sandwiched with a Parafilm membrane. (III) Leaf feeding assay for DcRV delivery via the plant. DcRV-infected *D. citri* adults were confined in pipe-like cages and fed on a clean leaf for 8 days. Uninfected *D. citri* adults were fed on the same feeding zone as infected insects for 10 days. The yellow dotted circle indicates the feeding zone, and the red dotted circle indicates the distal part. (B) DcRV could not infect the orange jessamine plants. (I) Schematic illustration of the preparation for tested leaves. (II) RT-PCR analyses showing the absence of DcRV infection in orange jessamine plants. Total RNA of leaves were extracted and tested for the presence of a partial region of DcRV S8 using PCR. Lane M, DNA ladder; lane 1, leaves of healthy control plants; lane 2, DcRV-infected *D. citri*; lane 3, feeding leaves; lane 4, new leaves on the flush shoots of the plant that was fed on by DcRV-infected *D. citri*. (C) The relative accumulation of P8 RNA in organs of DcRV-injected California *D. citri* insects was detected using RT-qPCR assays. The results were normalized against the level of actin mRNA, and the accumulation level of P8 at 1 dpi was normalized as 1. Means (±SD) from 3 biological replicates are shown. (D) Vertical transmission of DcRV to offspring. In the parent *D. citri* insect, (−) means uninfected Californian insects and (+) means DcRV-infected Hawaiian insects. *N* is the sample size for each treatment. Data displayed here are representative of 3 replicates.

To understand the manner of DcRV transmission, we assessed the DcRV incidence in our caged *D. citri* organisms (Hawaiian population). RT-quantitative PCR (RT-qPCR) assays showed 100% of *D. citri* insects were infected, and the mean viral genome copies in adults of infected *D. citri* insects was 5.65 × 10^8^ copies/μg RNA (see Data Set S1 in the supplemental material). The field-collected *D. citri* insects collected in Hawaii had mean viral genome copies of 1.25 × 10^8^ copies/μg RNA (Data Set S1). To determine whether DcRV could be horizontally transmitted among *D. citri* insects, we next used oral and injection approaches in attempts to transmit DcRV. Because the Hawaiian population of *D. citri* was 100% DcRV infected, we used the California *D. citri* population, which we first determined to be DcRV free, as the recipient. We first used membrane feeding using California *D. citri* with a crude DcRV virion preparation (Fig. 2AI). Second, we allowed uninfected California *D. citri* and DcRV-infected Hawaii *D. citri* insects to feed through a shared artificial diet, a “kiss model,” for 5 days (Fig. 2AII). For both assays, the recipient California *D. citri* insects were transferred to healthy citrus plants for 5 days. RT-qPCR analysis showed that none of the recipient *D. citri* insects acquired DcRV (Table 2).

**TABLE 2:** Percentage of individual *D. citri* organisms that acquired DcRV after oral acquisition or hemolymph injection

Horizontal transmission	Method	Insects (%) that acquired DcRV^*a*^		
		I	II	III
Oral acquisition	Membrane feeding	0	0	0
	Kiss model	0	0	0
	Leaf feeding	0	0	0
Hemolymph injection	Microinjection	100	100	100

a Twenty insects were used in each experiment.

As a final attempt to determine whether *D. citri* could be infected via oral acquisition, leaf feeding assays were performed (Fig. 2AIII). DcRV-infected Hawaii *D. citri* insects were caged on leaves of healthy citrus plants for 8 days. RT-qPCR analysis showed that DcRV could be detected from the feeding zone of the leaf, but the mean viral genome copies was 10^3^-fold lower than that in adults of infected *D. citri* insects (data not shown). No DcRV could be detected in the distal parts of the same plants (data not shown), suggesting that DcRV did not move within plant tissues from the location where it was deposited. Twenty uninfected California *D. citri* insects next were confined for 10 days on the same feeding zone as that used by the DcRV-infected Hawaii *D. citri* insects. RT-qPCR assays demonstrated that 68% of *D. citri* insects contained no detectable DcRV. However, 32% were DcRV positive, but the number of mean viral genome copies was 10^7^-fold lower than that in adults of the DcRV-infected Hawaii *D. citri* insects (data not shown). The DcRV-positive California *D. citri* insects were allowed to oviposit on plants, and adults of the next generation were found to be DcRV negative (data not shown). These results suggest that the very low DcRV titers detected by RT-qPCR immediately after feeding most likely were due to DcRV picked up by the California *D. citri* insects feeding on leaves, likely residual virions in the alimentary canal. Taken together, these data strongly suggest that the California *D. citri* insects could not be infected by DcRV via oral acquisition. It was also revealed that DcRV could not infect the Citrus macrophylla plant.

To determine whether DcRV could infect other plants, the orange jessamine (*Murraya paniculata* L.), another species that is closely related to *Citrus* and which is a preferred host for *D. citri*, was chosen. DcRV-infected *D. citri* insects were allowed to feed on the new leaves on flush shoots of orange jessamine plants (Fig. 2BI). The result from RT-PCR detection showed that both the new and feeding leaves were DcRV negative (Fig. 2BII), indicating that DcRV could not infect the orange jessamine plants.

As a final attempt to infect California *D. citri* with DcRV, we performed intrathoracic injection into California *D. citri* insects using a crude DcRV extract from Hawaii *D. citri*. At 5 days postinoculation (dpi), the organs of the injected *D. citri* insects, including the alimentary canal and reproductive systems, were dissected and analyzed by RT-qPCR. These results showed that level of the *P8* gene increased 44-fold (Fig. 2C), suggesting that California *D. citri* insects were susceptible to DcRV and could be infected by microinjection. At 10 dpi, the relative accumulation levels of the *P8* gene increased up to 171-fold (Fig. 2C). We subsequently monitored this DcRV-injected colony for 20 generations and found that the infection rate of each generation was 100%, showing that DcRV could stably infect California *D. citri* for generations. Taking these results together, we confirmed that DcRV was infectious and infected both Californian and Hawaiian *D. citri* but was not orally acquired.

DcRV incidence of 100% in DcRV-infected colonies suggested that it must be readily transmitted vertically to progeny. To test this, approximately 200 5-day-old eggs laid by 15 individual DcRV-infected females were tested by RT-qPCR. All of the eggs were DcRV positive, suggesting that DcRV had infected eggs before they hatched. To test whether DcRV could be transmitted sexually by male and/or female *D. citri* insects, mating experiments were conducted using the following mating pairings: female (uninfected Californian) × male (uninfected Californian), female (infected Hawaiian) × male (uninfected Californian), female (uninfected Californian) × male (infected Hawaiian), and female (infected Hawaiian) × male (infected Hawaiian). The insects were held together for 7 days (Fig. 2D). The DcRV incidence in progeny of DcRV-infected females was 100%, regardless of whether males were DcRV infected or uninfected. In contrast, when DcRV-infected males were mated with healthy females, DcRV incidence among the progeny was only 8.2% (Fig. 2D). These results suggest that DcRV was biparentally sexually transmitted but the maternal transmission was dominant, while paternal transmission was a supplementary means for viral vertical transmission.

### P10 of DcRV is a tubule-forming, virion-packaging protein.

**FIG 3:**
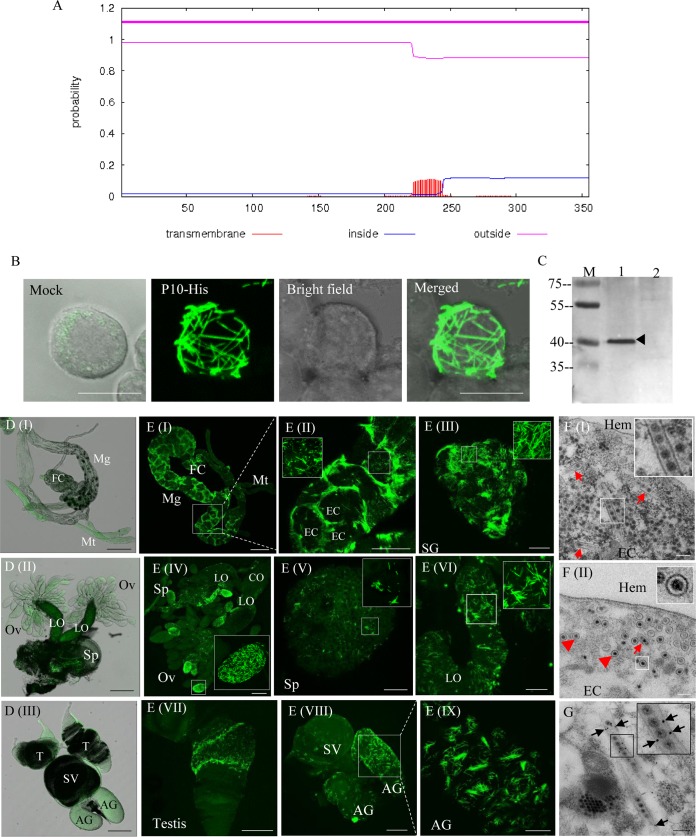
DcRV P10 is associated with tubules that can package virions in DcRV-infected *D. citri*. (A) Prediction of hydrophobic regions for DcRV P10 generated with TMHMM. (B) Expression of P10 fused with a His tag showing filament-like structures in recombinant baculovirus-infected Sf9 cells at 72 hpi. Sf9 cells were treated with fluorescein isothiocyanate-conjugated His-6×His tag antibody. The image of mock infection with green fluorescence (His-FITC antibody) was merged under a background of transmitted light. Bar, 10 μm. (C) Western blot analyses showing the specificity of antibodies against the P10 peptide. Total proteins of Californian DcRV-infected and uninfected *D. citri* insects were separated by SDS-PAGE and analyzed with P10 antibodies. Lane M, protein ladder; lane 1, protein extracts from DcRV-infected *D. citri*; lane 2, protein extracts from uninfected *D. citri*. (D) Immunofluorescence microscopy showing the absence of P10 in the alimentary canal (I), female reproductive system (II), and male reproductive system (III) of uninfected *D. citri*. The images with green fluorescence (P10-FITC antibody) were merged under a background of transmitted light. Bars, 200 μm. (E) Immunofluorescence microscopy showing that P10 widely distributes in the body of DcRV-infected California *D. citri*. The internal organs of DcRV-infected *D. citri* were treated with P10-FITC antibody (green) and examined by confocal microscopy. Images are presented in stacked sections. Insets are enlarged images of the boxed areas in each panel. Bars are 200 μm (I), 100 μm (II, IV, VII, and VIII), 20 μm (VI and IX), and 50 μm (III and V). (F) Electron micrographs showing virion-containing tubules in the cytoplasm of epithelial cells of guts. Insets are enlarged images of the boxed areas. Red arrows or arrowheads indicate the longitudinal or transverse sections of tubules packaging DcRV virions. Bars, 200 μm. (G) Immunogold labeling of P10 on virion-containing tubules in the cytoplasm of epithelial cells of guts. Black arrows mark gold particles. The inset is an enlarged image of the boxed area. Bars, 200 μm. Images are representative of the results of more than 3 experiments. FC, filter chamber; Mg, midgut; Mt, Malpighian tubule; EC, epithelial cell; SG, salivary gland; Ov, ovary; Sp, spermatheca; LO, lateral oviduct; CO, common oviduct; AG, accessory gland; SV, seminal vesicle; Hem, hemolymph.

Because most plant-infecting reoviruses encode a nonstructural protein that is hydrophobic and forms tubules containing virions, we attempted to determine whether DcRV encoded a protein with similar properties. Previous studies showed that the amino acid sequence of DcRV P10 encoded by S10 was similar to NLRV P10 at a low level (similarity of 24%) (3). However, whether or not these two proteins function similarly could not be predicted. Further BLASTp analysis for NLRV P10 indicated that the tubule-associated protein P7-1 of *Southern rice black-streaked dwarf virus* (SRBSDV) (12–14) in the genus *Fijivirus* was the closest protein, with 24% identity. Different transmembrane (TM) prediction tools were used to analyze the presence of the hydrophobic regions in DcRV P10, and the predicted results varied according to the methods used. The DAS Transmembrane Prediction server (15) predicted two TM regions (data not shown), whereas TMHMM identified only one (Fig. 3A). Thus, DcRV P10 was a putative transmembrane protein.

We next used the Bac-to-Bac baculovirus expression system to express P10 in Sf9 cells. Sf9 cells were inoculated with recombinant baculovirus that encoded P10 fused with a His tag. As seen by immunoﬂuorescence microscopy, at 48 h postinfection (hpi) the P10 protein was associated with filament-like structures (Fig. 3B), corresponding in size and morphology to the expression of tubule-associated protein of plant-infecting reoviruses in Sf9 cells (12–14).

We used antibodies prepared to P10 and immunoblots in attempts to identify P10 in DcRV-infected *D. citri*. P10 antibodies recognized a 40-kDa protein, consistent with the predicted molecular weight of P10, in protein extracts from DcRV-infected but not uninfected Californian *D. citri* (Fig. 3C). To study the distribution of P10 in the body of DcRV-infected Californian *D. citri*, immunofluorescence microscopy was performed via immunolabeling using IgG of P10, which was conjugated directly to fluorescein isothiocyanate (P10-FITC). The results showed that no specific fluorescence was detected in the organs of uninfected Californian *D. citri* (Fig. 3D). In contrast, in DcRV-infected *D. citri*, P10 was present in filament-like structures similar in morphology to those seen in Sf9 cells infected with the recombinant baculovirus that encoded P10. The filament-like structures were widely distributed within different organs of *D. citri* (Fig. 3E), similar to what has been observed for the tubules of plant-infecting reoviruses in their insect vectors (12–14). In the gut, tubule-like structures clearly labeled the outline of epithelium (Fig. 3EI and II). In the reproductive system of DcRV-infected *D. citri*, P10 was localized in the ovary, spermatheca, oviduct, testis, accessory gland, and seminal vesicle (Fig. 3EIV to IX). To further examine the tubule-like structures, TEM was used to analyze thin sections from DcRV-infected *D. citri*. This showed that the tubule structures were approximately 90 nm in diameter and distributed in the cytoplasm of epithelial cells of guts (Fig. 3FI). Within these tubule structures, virus-like particles of 70 nm in diameter were present (Fig. 3FI). We also observed the transverse sections of tubules of about 90 nm in diameter with virions inside the epithelial cells of guts (Fig. 3FII). The observation for DcRV-infected Hawaiian *D. citri* showed the same results of immunofluorescence and TEM as those for DcRV-infected Californian *D. citri* (data not shown). Further immunoelectron microscopy showed that P10-specific IgG specifically recognized the virion-containing tubule structures (Fig. 3G), confirming that DcRV P10 had an ability to form tubular structures.

Taking these findings together, DcRV P10 in DcRV-infected *D. citri* shows a morphology and distribution similar to that seen for the tubule-forming proteins of plant-infecting reoviruses in their insect vectors (12–14). Thus, it may be possible that DcRV-encoded P10 had similar functions, such as those for viral intercellular spread.

### P10 tubules carry DcRV virions to the ovary for transovarial transmission.

**FIG 4:**
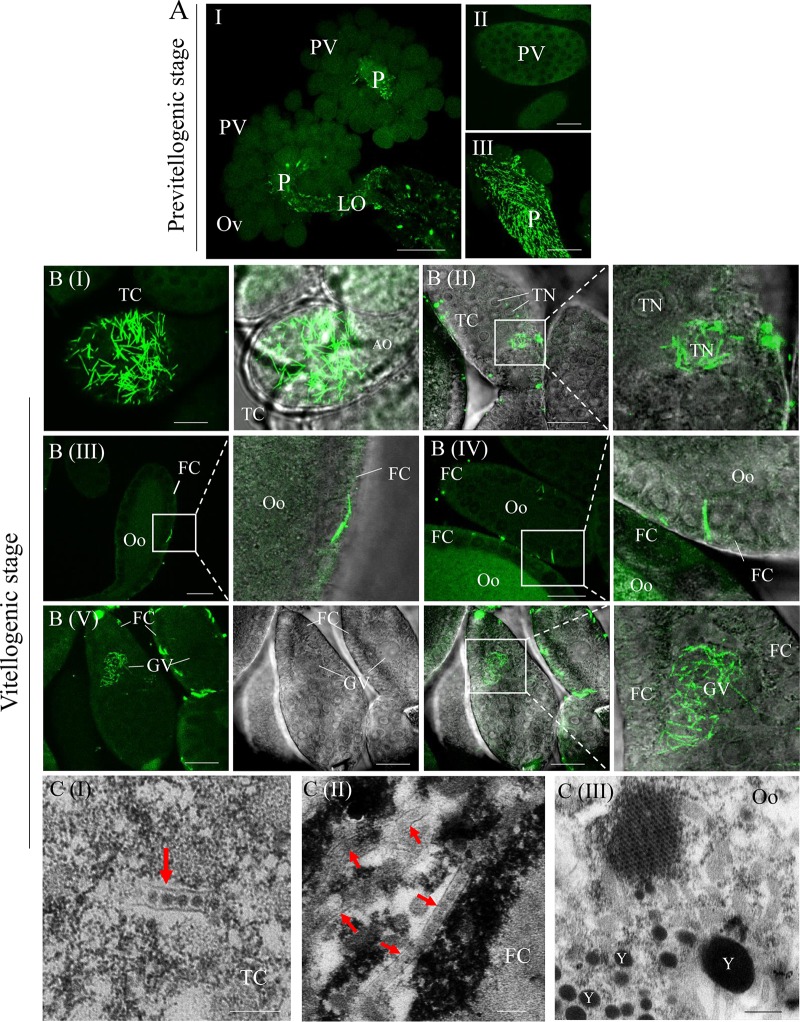
P10 tubules carry DcRV virions into the ovary at the vitellogenic stage. (A) Immunofluorescence microscopy showing that P10 tubules fail to localize to the ovarioles at the previtellogenic stage. Bars are 100 μm (I), 20 μm (II), and 50 μm (III). (B) Immunofluorescence microscopy showing the distribution of P10 tubules in the trophic chamber and oocyte-developed ovarioles at the vitellogenic stage. The internal ovaries of DcRV-infected *D. citri* insects were immunolabeled with P10-FITC (green) and then examined by confocal microscopy. Images are presented in stacked sections. The enlarged images display green fluorescence (P10-FITC) and bright fields of the merged images of the boxed areas in each panel, indicating the P10 distribution in the ovary. Bar, 20 μm. (C) Electron micrographs showing virion-containing tubules (arrows) in the vitellogenic ovary. Insets are enlarged images of the boxed areas in each panel. Bars are 200 nm (I and II) and 400 nm (III). Ov, ovary; PV, previtellogenic ovariole; P, pedicle; LO, lateral oviduct; TC, trophic chamber; AO, arrested oocyte; TN, trophocyte nuclei; FC, follicular cell; Oo, oocytes; GV, germinal vesicle; Y, yolk. All micrographs are representative of at least 3 replicates.

Because our data suggest that P10 could form virion-containing tubules that have the putative function for viral intercellular spread, we next investigated whether P10 could also play a role in viral transovarial transmission of DcRV to progeny. We focused on P10 expression and distribution in the ovaries with the development of the reproductive system of DcRV-infected California *D. citri*. Ovaries of newly emerged adults showed small ovarioles and oocytes, which were at the previtellogenic stage (Fig. 4A). Immunofluorescence microscopy showed abundant P10 tubules localized in the epithelial cells of pedicles and oviducts, but no immunolabeling was detected on the previtellogenic ovarioles (Fig. 4A). In contrast, in mature females, ovarioles at the vitellogenic stage were completely developed into two regions, the trophic chamber and vitellarium (16, 17) (Fig. 1AI). The trophic chamber was syncytial and contained several trophocyte nuclei. The oocyte, located in the center of the vitellarium, was surrounded by a single layer of follicular cells on its surface (Fig. 4B). Immunofluorescence microscopy demonstrated that numerous P10 tubules accumulated in the cytoplasm of the trophic chamber, some of them localized in the trophocyte nuclei (Fig. 4BI and II). In the vitellarium, some P10 tubules localizing in the follicular cells penetrated to the adjacent cells or inserted in the gap between follicular cells and penetrated to the oocytes (Fig. 4BIII and IV). We even observed P10 tubules in germinal vesicles, which were present in the center of oocytes (Fig. 4BV). The close association of P10 tubules containing DcRV virions with mature ovaries suggested that P10 tubules provide access for DcRV transovarial infection at the vitellogenic stage.

To confirm our observations, TEM was conducted to visualize the P10 tubules in the vitellogenic ovaries of DcRV-infected *D. citri*. In the cytoplasm of the trophic chamber, tubules of approximately 90 nm in diameter, with viral particles inside, were observed (Fig. 4CI). Virion-containing tubules were also detected in the follicular cells of vitellarium (Fig. 4CII), consistent with the observations from immunofluorescence microscopy (Fig. 4B). We also determined the presence of free virions of approximately 70 nm in diameter or aggregated into crystalline arrays within the cytoplasm of oocytes (Fig. 4CIII), suggesting the viral infection of oocytes and, thus, that offspring can be infected at the oocyte stage. All of these observations indicated that DcRV particles could be accompanied by P10 tubules to enter the ovary for transovarial transmission.

### Knockdown P10 suppresses viral accumulation in eggs.

**FIG 5:**
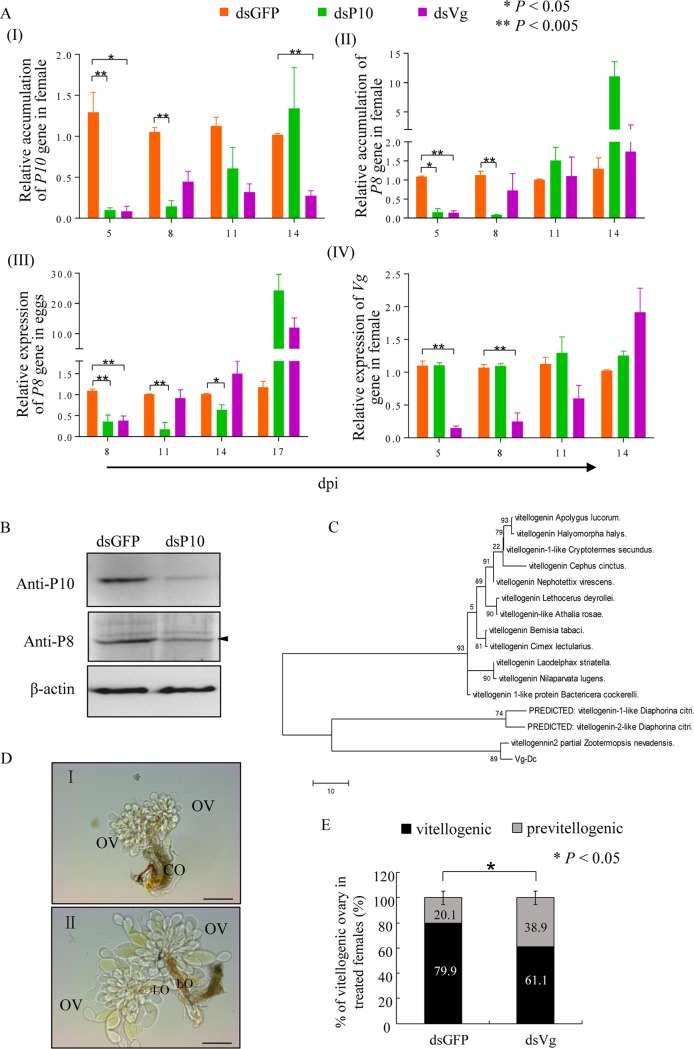
Knockdown of P10 decreases the accumulation of DcRV in *D. citri* female ovaries and suppresses DcRV infection of eggs. (A) RT-qPCR of relative gene accumulation of P10, P8, and Vg in ovaries of dsRNA-treated *D. citri* females or P8 in eggs laid by dsRNA-treated *D. citri* females. Means (±SD) from more than 3 biological replicates are shown. *, *P* < 0.05; **, *P* < 0.005. Data were analyzed with a two-tailed *t* test in GraphPad Prism 7. (B) At 7 dpi, expression of P10 and P8 proteins in ovaries of females were decreased caused by dsP10, as revealed by Western blotting with P10- or P8-speciﬁc IgGs. β-Actin was used as a loading control. Data shown here are representative of 3 replicates. (C) Phylogenetic relationships of Vg orthologs of *D. citri* with counterparts from other insects. The available amino acid sequences were aligned using the ClustalW program, and phylogenetic trees were reconstructed by maximum likelihood analysis using MEGA 5.1. Reliability of the phylogenetic trees was estimated by calculating bootstrap confidence limits based on 1,000 replicates. (D) Transmitted light micrograph of previtellogenic (I) and vitellogenic (II) ovaries of *D. citri* insects. Ov, ovary; LO, lateral oviduct; CO, common oviduct. Bars, 200 μm. (E) The percentage of vitellogenic ovaries in dsRNA-treated females at 6 dpi. Means (±SD) from three biological replicates are shown. *, *P* < 0.05. Data were analyzed with a one-tailed *t* test in GraphPad Prism 7.

In attempts to further verify the critical role P10 tubules played in viral infection of oocytes, we attempted to determine the effects of RNAi knockdown of P10 accumulation in DcRV-infected female ovaries and the effects on DcRV transovarial transmission. Newly emerged uninfected California female adults were injected with a mixture of DcRV and dsRNAs targeting the DcRV *P10* gene (dsP10) or DcRV plus dsRNAs for the green fluorescent protein (GFP) coding sequence (dsGFP). Compared with the dsGFP control group, the reduction of P10 RNA (from 93% to 47%) in dsP10 treatment from 5 to 11 dpi indicated that RNAi effects induced by dsP10 were triggered and active in ovaries of injected females (Fig. 5AI). However, the significant decrease of gene accumulation of P8 (from 94% to 86%) in dsP10 treatment from 5 to 8 dpi, in contrast to the dsGFP group (Fig. 5AII), also revealed that injection of dsP10 not only suppressed P10 accumulation but also suppressed DcRV accumulation in ovaries. Western blot analysis was used to confirm the reduced protein accumulation of P10 and P8 induced by RNAi targeting P10 at 7 dpi (Fig. 5B). The DcRV accumulation in the eggs from these dsRNA-treated females also demonstrated significantly reduced accumulation (from 83% to 38%) from 5 to 14 dpi (Fig. 5AIII). These results suggested that knockdown of P10 induced by dsP10 introduction suppressed DcRV accumulation and efficient spread in ovaries of females and also inhibited the virus titers that were subsequently passed to the next generation.

Vitellogenin (Vg), the precursor protein of egg yolk, deposits in the oocytes for the rapid growth of oocytes (18). Thus, Vg is a critical protein associated with oocyte development and serves as a physiological indicator of vitellogenesis. To gain insight into the importance of the vitellogenic stage for P10 entering the ovary, a predicted partial vitellogenin-1-like gene sequence of *D. citri* (accession number XM_008488883) was found in GenBank. On the basis of this finding, a fragment of about 2,000 nt in length was generated by using RT-PCR. The phylogenetic analysis showed that the amino acid sequences of Vg (Vg-Dc) clustered with those of other insect species (Fig. 5C).

We next attempted to manipulate the expression of Vg in newly emerged female adults by RNAi. Uninfected California female adults were injected with the mixture of DcRV and dsRNAs targeting Vg (dsVg). Time course experiments showed the decreased gene accumulation of Vg by 47% to 87% in female ovaries from 5 to 11 dpi in dsVg treatment compared with that of the dsGFP group (Fig. 5AIV). This indicated that the RNAi specific for Vg was triggered and active. At 6 dpi, the dissected ovaries displayed a greatly reduced percentage of vitellogenic ovaries in dsVg treatment, in contrast to the dsGFP control group (Fig. 5D and E). This result demonstrated that knocking down Vg accumulation delayed the development of ovaries, indicating that the Vg fragment we obtained was probably a part of the *Vg* gene sequence. We next assessed the accumulation of the DcRV P8 and P10 coding sequences in the ovaries from dsVg-treated *D. citri*. The P10 coding RNA was reduced from 5 to 14 dpi, while ovaries from dsVg-treated insects only showed reductions from 5 to 8 dpi for the P8 coding RNA (Fig. 5AI and II). The DcRV accumulation in eggs laid by dsVg-injected females demonstrated significant downregulation at 8 dpi (Fig. 5AIII). The accumulation then gradually increased with the RNAi effects decreasing in subsequent days (Fig. 5AIII), displaying a gene accumulation tendency identical to that of P8 in dsVg-treated female ovaries. The similar gene accumulation level of Vg in dsP10 treatment and the dsGFP control group revealed that the inhibition of P10 possibly had no substantial effect on Vg deposition and oocyte development (Fig. 5AI). Taken together, the dsVg treatment not only delayed the development of ovaries but also inhibited the viral accumulation in eggs, revealing that the vitellogenic stage was important for viral infection in oocytes. Because we observed virion-containing P10 tubules in oocytes at the vitellogenic stage, we suggest that the vitellogenic stage is important for P10 entering the oocyte. Therefore, we confirmed that P10 was associated with delivery of viral particles to the oocytes at the vitellogenic stage, resulting in eggs that were DcRV infected before fertilization.

### Virion-packaging P10 tubules associate with sperm for viral paternal transmission.

While DcRV-infected females showed the greatest DcRV transovarial transmission rate, we also showed that DcRV-infected Hawaiian males, when mated with uninfected Californian females, could contribute to a low percentage of DcRV vertical transmission. Therefore, to address the mechanism behind the paternal transmission of DcRV, we assessed the DcRV infection in female adults collected from uninfected Californian *D. citri* insects and caged with DcRV-infected Hawaiian males for 7 days. At 8 days postcaging, immunofluorescence microscopy failed to detect P10 in the reproductive systems of females (data not shown). However, at 30 days postcaging, the ovaries of approximately 20% of females were immunolabeled with P10 (data not shown), indicating that females became infected by DcRV after mating with DcRV-infected males.

**FIG 6:**
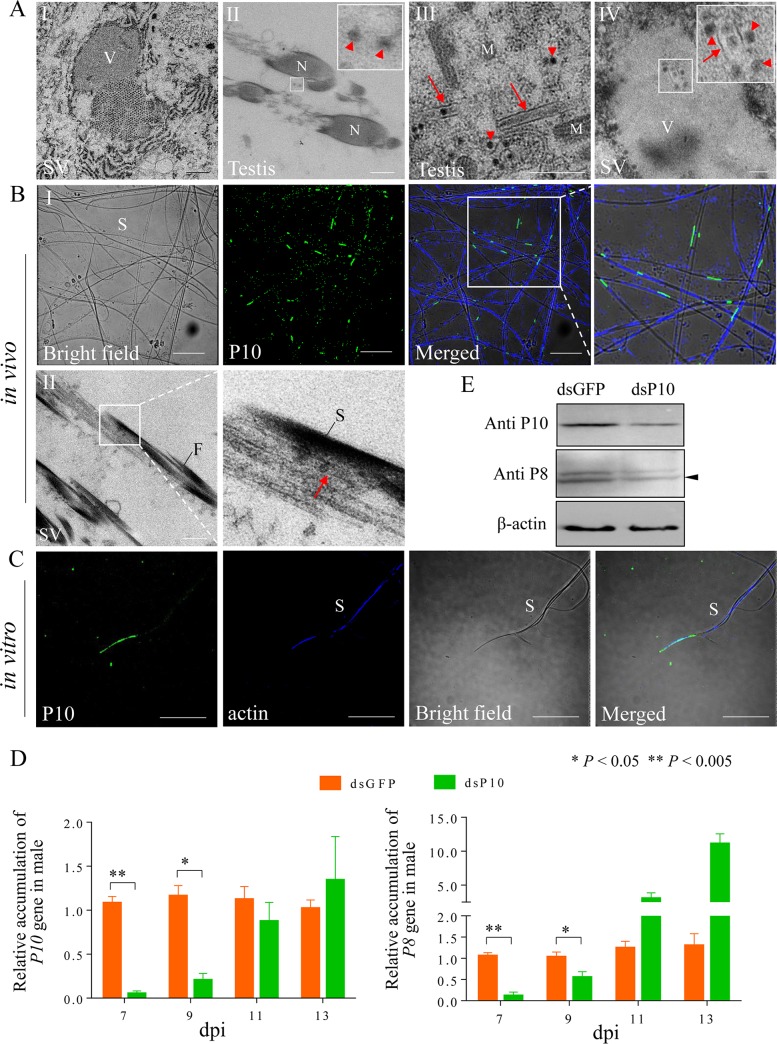
P10 tubules associate with sperm of *D. citri* males. (A) Electron micrographs showing DcRV infection and virion-packaging tubules in the epithelial cells of testes and seminal vesicles of *D. citri* males. Insets are the enlarged images in the boxed areas in each panel. Bars are 1 μm (I), 500 nm (II and III), and 200 nm (IV). (B) Immunofluorescence microscopy (I) and electron micrographs (II) showing virion-containing P10 tubules associated with *D. citri* sperm. For the immunofluorescence microscopy, the sperm samples of DcRV-infected Californian *D. citri* males were immunolabeled with P10-FITC (green) and actin dye phalloidin-Alexa Fluor 647 carboxylic acid (blue) and then examined by confocal microscopy. Images are presented in single sections. The enlarged images display green fluorescence (P10-FITC), blue fluorescence (actin), and bright fields of the merged images, indicating P10 associated with sperm. Bars are 20 μm (I) and 500 nm (II). (C) Immunofluorescence microscopy showing P10 associated with sperm *in vitro*. The sperm of uninfected Californian *D. citri* males were incubated with P10-containing DcRV crude extracts from *D. citri* females *in vitro*. The samples were immunolabeled with P10-FITC (green) and actin dye phalloidin-Alexa Fluor 647 carboxylic acid (blue) and then examined by confocal microscopy. Images are presented in single sections. Bar, 20 μm. Arrows or arrowheads indicate the virion-packaging tubules or viral particles. V, viroplasm; SV, seminal vesicle; N, nucleus; M, mitochondrion; S, sperm; F, ﬂagellum. (D) RT-qPCR of relative gene accumulation of P10 and P8 in testes of dsRNA-treated *D. citri* males. Means (±SD) from 3 biological replicates are shown. *, *P* < 0.05; **, *P* < 0.005. Data were analyzed with a two-tailed *t* test in GraphPad Prism 7. (E) At 7 dpi expression of P10 and P8 proteins in testes of males was decreased by dsP10, as revealed by Western blotting with P10- or P8-speciﬁc IgGs. β-Actin was used as a loading control. Data shown are representative of 3 replicates.

Because P10 tubules played an important role in viral maternal transmission, we assessed whether P10 also participated in the DcRV paternal transmission. The reproductive systems of DcRV-infected males were dissected and analyzed by TEM. Within the epithelial cells of testis and seminal vesicles, numerous 70-nm-diameter viral particles were found concentrated in the cytoplasm, distributed at the periphery of viroplasms, or aggregated into crystalline arrays (Fig. 1AVIII and 6A). Some free virions were scattered at the periphery of sperm in the lumen of testis and seminal vesicles (Fig. 6A), indicating that DcRV could infect the intact testis and seminal vesicles. We then used immunofluorescence microscopy and found that P10 also was localized in testis, seminal vesicles, and accessory glands of infected Californian *D. citri* insects (Fig. 3EVII and VIII). TEM confirmed that in these organs, the virus-containing P10 tubules were in the cytoplasm of epithelial cells, which also contained amounts of mitochondria, endoplasmic reticulum, and vesicles (Fig. 6AIII). Some tubules were also found in the viroplasms within the epithelial cells (Fig. 6AIV). Because the sperm moves from male to female during mating, we next investigated the association of P10 with sperm. The sperm samples were extracted from the testis and seminal vesicles. Examination by bright-field microscopy showed the sperm as long and threadlike (Fig. 6BI), consistent with the reported morphology of sperm (19). Immunofluorescence microscopy showed that approximately 20% of the sperm cells had P10 tubules (Fig. 6BI). TEM also confirmed that virion-packed tubules were associated with sperm (Fig. 6BII). The observation for Hawaiian DcRV-infected *D. citri* showed the same results of immunofluorescence and transmission electron microscopy as the infected California *D. citri* insects. To clarify whether the P10 tubules have an inherent ability to bind the sperm, the sperm of uninfected males was incubated *in vitro* with P10-containing crude extract obtained from DcRV-infected females. Immunoﬂuorescence microscopy allowed us to locate P10 speciﬁcally on sperm (Fig. 6C). Thus, P10 tubules were able to associate with sperm, which might provide access for viral paternal transmission.

We attempted to manipulate the accumulation of P10 in DcRV-infected males by using RNAi. California male adults were injected with a mixture of DcRV crude extract plus dsP10 or dsGFP. This resulted in decreased P10 gene accumulation (by 23% to 95%) in dsP10 treatment from 7 to 11 dpi (Fig. 6D). The significant reduction of gene accumulation of P8 (from 90% to 48%) from 7 to 9 dpi caused by dsP10 introduction was consistent with the results in ovaries (Fig. 6D), suggesting that the RNAi effects resulted in decreased DcRV accumulation. Western blot analysis confirmed the downregulated accumulation of P10 and P8 proteins in dsP10 treatment at 7 dpi (Fig. 6E). Most interesting was that the infection rate of offspring after dsP10 treatment in males resulted in no transovarial transmission after the treated males mated with uninfected females. In contrast, the dsGFP treatment in males resulted in approximately 4% of offspring being infected (Table 3). Thus, injection of dsP10 led to the inhibition of DcRV accumulation in testes and the subsequent failure of DcRV paternal transmission. These results confirmed that virus-packaging P10 could exploit the motile sperm as a vehicle via direct and inherent association to facilitate the viral paternal transmission.

**TABLE 3:** Detection of DcRV in the offspring of P10-knockdown DcRV-infected *D. citri* males mating uninfected females

Expt no.	Detection [% (no. infected/no. tested)] of:	
	dsGFP^*a*^	dsP10
I	4.3 (1/23)	0 (0/20)
II	4.0 (1/25)	0 (0/22)
III	4.3 (1/23)	0 (0/23)

a *P* < 0.005.

## DISCUSSION

### A novel insect reovirus.

In a previous study, our laboratory identified sequences of unknown viruses in global populations of *D. citri* using small RNA and transcriptome sequencing (3). We hypothesized that these sequences were representative of infectious viruses, and here we determined that DcRV is an infectious virus and was transmissible via injection to naive *D. citri* insects. We identified virions (Fig. 1) showing the morphology and structural characteristics of viruses in the *Reoviridae* (5). Phylogenetic comparisons suggested DcRV was most similar to NLRV, a tentative member of the genus *Fijivirus* (3). Most viruses in the genus *Fijivirus* are dual-host viruses, infecting the insect vectors as well as plants hosts. However, NLRV is so far only known to infect planthoppers, specifically *Nilaparvata lugens* Stål (Heteroptera: Delphacidae), but the virus can move through rice plants without replicating and then be acquired by and infect healthy *N. lugens* insects feeding on other parts of the same plant (6–8). We found no evidence that DcRV could infect citrus and/or orange jessamine plants, and transmission experiments showed that although DcRV was delivered by DcRV-infected Hawaiian *D. citri* to the plants via feeding, it could not replicate and/or move in leaves (Fig. 2A and B). Furthermore, DcRV was not transmissible to naive Californian *D. citri* insects via oral acquisition (Table 1) but was transmissible by injection and via sexual transmission, and it was maintained in the population by 100% transovarial transmission to progeny. In our studies, DcRV showed no obvious pathogenicity to infected *D. citri*; thus, it might be an insect-specific virus, such as *Leafhopper A virus* (LAV), *Peregrinus maidis virus* (PgMV), *Drosophila S virus* (DSV), and NLRV (7, 20).

### Transovarial transmission in vertical transmission.

Vertical transmission is a crucial strategy for the persistence of viruses in insect populations. This can involve viral integration into the host genome, maternal transmission through eggs, and/or paternal transmission through sperm (21). For the vertical transmission of viruses, all the sigma viruses and DSV in *Drosophila* have been found to be passed to progeny by biparental transmission (22–24). The reovirus-like Oniscoidea masculinizing virus can also be vertically passed from both females and males to the next generation (25). We showed here that DcRV was transmitted in a biparental manner (Fig. 2). We found 100% transovarial transmission from DcRV-infected females, and because DcRV can be transmitted via DcRV-infected males to uninfected females, this serves as the means for spread into uninfected *D. citri* populations.

Maternal transmission of insect viruses involves transovarial transmission and transovum transmission. The former refers to the inheritance resulting from mother-originating virus infecting the developing oocytes. This has been shown for several viruses, including orthobunyaviruses, insect-specific flaviviruses, and mosquito-transmitted arboviruses (26–28). In contrast, transovum transmission refers to the mother-originating viral passage through contamination of the exterior egg surface. This has been shown for some viruses, including the Lepidoptera-infecting nuclear polyhedrosis virus (29–31). NLRV, to which DcRV was most closely phylogenetically related, was shown to be passed in a transovum manner (7). Here, we found DcRV was transovarially transmitted. DcRV-infected mothers could give 100% infection in the progeny. This ability of DcRV transovarial transmission appears to be one of the greatest among the known insect-borne viruses.

During viral transovarial transmission in insects, the important processes are the invasion of viral particles into the oocytes and the production of infectious virions, resulting in infection of the unfertilized egg (14, 32). In some examples virions appear to be carried by the insect protein Vg to infect the germarium or oocytes of ovarioles. This has been shown for *Rice stripe virus* (RSV) and *Tomato yellow leaf curl virus* (TYLCV) (33–35). Viruses can also employ inclusion bodies formed by virus-encoded nonstructural proteins to infect oocytes, as has been shown for *Rice gall dwarf virus* (RGDV) (36). In this study, by using immunofluorescence microscopy and transmission electron microscopy, we found that P10 tubules carried virions to oocytes (Fig. 4). When we knocked down P10 by RNAi in females, this not only suppressed the viral spread and accumulation but also reduced the viral accumulation in eggs (Fig. 5). It is necessary to mention that the relative accumulation of P10 and P8 genes recovered to normal levels at 14 days after dsP10 treatment (Fig. 5A). The relative expression of the Vg gene also showed a similar increase at 14 days after dsVg treatment (Fig. 5A). These increases of relative accumulation of target genes likely reflect the recovery of virus replication as the RNAi effects wear down over time. We found that in the dsVg treatment, while the relative accumulation of P10 was still reduced after 8 dpi, the relative accumulation of P8 in ovaries and eggs was increased. It is assumed that some unknown compensation for the pathway of virus entering eggs is launched when P10 is inhibited, for example, when the free virions enter the eggs. We also found that the relative accumulation of P8 RNA showed a profile similar to that of P10 RNA in dsP10 treatment (Fig. 5A), suggesting that the knockdown of P10 expression not only blocked the formation of tubules but also affected overall DcRV replication/accumulation. These results once again provided evidence for the fact that each viral protein was essential for viral infection (13, 37). The same results were found for the relative accumulation of P8 RNA in eggs, indicating that knockdown of P10 in the females also affected the amount of DcRV passed from females to the eggs (Fig. 5A).

P10 appeared to contribute to transovarial transmission of DcRV during vitellogenesis. Based on the result that P10 served as a facilitator and safe vehicle for viral spread and secondary infection, virion-containing P10 tubules could not spread to the previtellogenic ovarioles first (Fig. 4A); thus, DcRV virions may not be able to infect ovarioles at previtellogenic stages. In general, at the vitellogenic stage, the period of rapid oocyte growth and yolk deposition, the follicular cells on the surface of the oocytes shrink (38). The gaps between follicular cells appear and provide free access of the hemolymph to the surface of the oocyte, because the only potential barrier, the tunica propria, is permeable to large molecules (38). In the present study, we found that this specific stage was utilized by the P10 tubules, which inserted into the gap and penetrated to the oocytes, for efficient viral delivery.

The Vg, forming the major egg yolk protein called vitellin, is mostly female specific, although small amounts are found in males (18). It is derived from fat bodies and ovarian follicular cells of female adults and preferentially taken up by the oocyte during vitellogenesis (39). Because Vg has the ability to enter the oocyte, it was unknown whether the oocyte-entering path of P10 tubules was associated with Vg deposition. However, the knockdown of Vg inhibited DcRV spread and accumulation in females and DcRV infection of eggs. This result highlights the important role of the vitellogenic stage for DcRV infection and P10 spread in oocytes.

### Paternal transmission.

To our knowledge, paternal transmission is less common among pathogens and endosymbionts of insects, and we know relatively little about it. Most bacteria are transmitted in a maternal mode, and relatively few viruses have been described to be transmitted via sperm (21, 40). The most well-documented paternal transmission is for sigma virus, a rhabdovirus infecting dipterans (31). The virions of Drosophila melanogaster *sigma virus* (DMelSV) are detected in sperm cells of adult Drosophila melanogaster insects (41), and here we found that DcRV can exploit sperm for paternal transmission. We showed that DcRV could be passed in relatively low efficiency from males to progeny for vertical transmission and even to females for horizontal transmission. This could be important for DcRV spread to naive populations, because we found no evidence for oral acquisition as a means of transmission. If a male *D. citri* mates with an uninfected female, DcRV will infect only about 8% of the offspring. However, once a female *D. citri* insect is DcRV infected, the virus will be passed to 100% of the next generation.

After mating, the sperm are stored and maintained in the spermatheca of the female (42). When the eggs pass down the oviduct, the sperm are released from the spermatheca for fertilization (42). The crossing experiments of female (Californian uninfected) × male (Hawaiian DcRV infected) and RNAi experiments revealed that postmating, DcRV initially failed to spread and infect the reproductive systems of females, but the eggs were infected. This suggests that the eggs were directly infected by DcRV-infected sperm during fertilization, whereas ovaries of approximately 20% of the females became infected at a later time after mating, indicating that the DcRV was from the infected sperm and invaded other tissues of the fertilized females.

When the details of DcRV paternal transmission were explored, we found DcRV virion-containing tubules in males. Virions packaged in P10 tubules were capable of binding sperm, resulting in DcRV infection of some progeny and the recipient females. It was unclear whether the association of P10 with sperm was physical binding or a specific protein-protein interaction, but it may represent a unique means for DcRV to utilize nonstructural proteins in male insects for vertical and horizontal transmission. Other viruses which are able to exploit the sperm for viral transmission are usually limited to the infection of sperm (41, 43, 44). The sperm-associated spread of DcRV via tubules might be less stable, because the exterior binding of tubules has a potential risk of loss and damage during mating. This might explain the reasons that the efficiency of DcRV paternal transmission was lower than that of other paternally transmitted viruses, such as the sigma viruses (23, 45). For the transovarial transmission, the spread of mother-originated DcRV and infection of oocytes occurred in the same individual, while for the paternal transmission, it was two different individuals that DcRV passed through, from the spread of sperm-associated P10 tubules to the viral delivery in females. The risk of loss and damage of P10 tubules from males to females was certainly higher than that in females, which may also cause the inefficiency of paternal transmission of DcRV compared with that of transovarial transmission.

### Tubule-associated proteins of reoviruses.

Many plant-infecting reoviruses encode proteins which assemble into virion-packaging tubular structures in virus-infected insect vectors (12–14, 46, 47). Because of their ability to allow for virion transport between cells, this efficient tool utilized by plant reoviruses overcomes the tissue and membrane barriers in the insect vector. For the NLRV, the virus most closely related to DcRV, it is unclear which, or if, any genome segments encode a protein that could form virion-containing tubules. In this study, we found that the P10 protein of DcRV, which is closest to the NLRV P10, possessed the properties of a tubule-associated protein, including the predicted hydrophobic region, and the capability of assembling into virion-containing tubular structures of approximately 90 nm in diameter (Fig. 3). The tubules formed by DcRV P10 were widely distributed in most organs of *D. citri* insects. It has been suggested that it is universal for insect-borne reoviruses to encode proteins which could assemble into virion-packaging tubular structures for viral spread in insects.

Besides tubules contributing to the viral intercellular spread in the epithelial cells, DcRV P10 tubules also were associated with the delivery of viral particles to the oocytes, which corresponds with the function of Pns11 tubules derived from the plant reovirus RGDV in leafhoppers (36). Thus, DcRV is the second virus which has been found to exploit a virus-encoded protein that assists in transovarial transmission. We also found that the oocyte-entering function of DcRV tubules was closely related to the development of ovaries (Fig. 4 and 5). We could not clarify whether the development of insects facilitates the viral spread or whether DcRV takes advantage of insect growth and development. DcRV tubules were associated with sperm for viral delivery in viral paternal transmission (Fig. 6). Among the reoviruses, so far no virus-encoded tubule-associated proteins have been found to have an ability to bind sperm. In conclusion, our results reveal a pivotal role for a nonstructural protein in biparental transmission of a novel insect virus.

## MATERIALS AND METHODS

### Insects and plants.

The uninfected colony of *D. citri* was started using field-collected *D. citri* insects from southern California, and the DcRV-infected colony of *D. citri* was started using field-collected *D. citri* insects collected in Hawaii. Both colonies were maintained separately on *Citrus macrophylla* plants in the Contained Research Facility (CRF) at UC–Davis.

### Antibody preparation.

The anti-P8 and anti-P10 polyclonal antibodies were prepared by Antibodies Incorporated (Davis, CA). Briefly, rabbits were immunized with the synthesized P8 peptide MKTTSPKNLYNISE or P10 peptide SDNNSNSLNVEEIDG 5 times to make anti-P8 or anti-P10 serum, respectively. IgG was purified from polyclonal antiserum using a protein A-Sepharose afﬁnity column.

To determine the speciﬁcity of the DcRV P10 antibodies, total proteins of DcRV-infected or uninfected Californian *D. citri* insects were extracted and separated by SDS-PAGE. Western blot analyses were performed with P10 antibodies to detect P10 protein.

The P8- or P10-specific IgG was conjugated directly to FITC (P8- or P10-FITC) according to the manufacturer’s instructions (Thermo Fisher Scientific).

### Electron microscopy.

For transmission electron microscopy, the internal organs of DcRV-infected Hawaiian *D. citri* insects were dissected and fixed in 2.5% glutaraldehyde in 0.1 M phosphate-buffered saline (PBS) at room temperature. Samples were secondarily fixed with 1% OsO_4_ in 0.1 M PO_4_ buffer for 2 h. Thereafter, dehydration was performed using ascending concentrations of acetone and then infiltrated and embedded in an epoxy resin mixture. Thin sections were cut and collected on 2- by 1-mm single-slot copper grids. The sections were stained with uranyl acetate, followed by detection under a JEOL-1230 transmission electron microscope in the Electron Microscopy Core Facility of UC–Davis. Uninfected California *D. citri* served as a technical control for the TEM assays.

For immunoelectron microscopy, sections were immunolabeled with the P8- or P10-specific IgG as the primary antibody, followed by treatment with goat anti-rabbit IgG conjugated with 10-nm-diameter gold particles as the secondary antibody (Abcam). Sections were detected under a transmission electron microscope (H-7650; Hitachi).

### Immunofluorescence microscopy.

Hemolymphs of 50 infected *D. citri* insects were collected and then were placed on Polysine adhesion slides (Thermo Fisher Scientific) and dried at room temperature. The samples were fixed in 4% paraformaldehyde for 2 h and treated with 0.2% Triton X-100 for 1 h as previously described (12) and then were immunolabeled with P8-FITC or actin dye phalloidin-Alexa Fluor 647 carboxylic acid (Thermo Fisher Scientific). The treated samples were examined with a Leica TCS SPE inverted confocal microscope.

Organs of *D. citri* insects were successively dissected, fixed, and treated with Triton X-100, as mentioned above. The samples then were immunolabeled with P10-FITC and finally detected using immunofluorescence microscopy.

### Membrane feeding.

To prepare the DcRV crude extracts for oral acquisition studies, approximately 0.03 g DcRV-infected Hawaiian *D. citri* insects were homogenized with 0.3 ml of the His-Mg buffer (0.1 M histidine, 0.01 M MgCl_2_, pH 6.2) and silicon dioxide. The slurry was clarified by centrifugation at 500 × *g* for 5 min and filtration through a 0.22-μm filter to ensure other nonviral pathogens were excluded.

The feeding diet contained the DcRV crude extract in 15% sucrose, and the feeding cage is shown in Fig. S2 in the supplemental material. Approximately 100 μl of feeding diet was sandwiched between Parafilm membranes, which covered one end of a small pipe-like cage (2.5 cm in diameter by 4 cm in height). Twenty adult *D. citri* insects were confined in each cage for 3 days, and the feeding cage was placed in a dark chamber with light from the upper side to attract the *D. citri* insects to the diet. The putative virus-acquiring *D. citri* insects were transferred to clean plants of *C. macrophylla* for 5 days. Three replicates were performed.

For the kiss model, the 15% sucrose solution was sandwiched between two cages for membrane feeding with Parafilm membranes. Twenty adults of infected Hawaiian or uninfected Californian *D. citri* insects were confined for 5 days in individual cages, and the diet was renewed every day. Three replicates were performed.

### Plant feeding.

Twenty adult DcRV-infected Hawaiian *D. citri* insects were confined for 8 days in a pipe-like cages (2.5 cm in diameter by 4 cm in height), of which one end was covered by mesh and one end was fixed on the underside of a *C. macrophylla* leaf. The DcRV-infected *D. citri* insects were removed, and 20 uninfected California *D. citri* insects were fed on the same feeding zone of the leaf for 10 days. These psyllids were then transferred to a clean *C. macrophylla* plant and allowed to reproduce. Three replicates were performed.

One cage containing DcRV-infected *D. citri* insects was placed on 3 plants of orange jessamine for one generation. These orange jessamine plants then were separated from *D. citri* insects. When the flush shoots grew, the new leaves on them and previously grown feeding leaves were collected for RT-PCR examination. The residual honey dew on the surface of feeding leaves was removed by washing with distilled water 3 times before testing. Three replicates were performed.

### Egg detection.

Approximately 200 5-day-old eggs laid by 15 individually reared females of the DcRV-infected Hawaiian colony were individually harvested. A TaqMan Gene Expression Cells-to-CT kit (Thermo Fisher Scientific) was used to lyse the eggs and detect the DcRV *P8* gene accumulation according to the manufacturer’s instructions.

### Hemolymph infection.

Approximately 80 uninfected Californian adult *D. citri* insects were microinjected at the intersegmental region of the thorax with the DcRV crude extract and then allowed to feed and reproduce on a healthy *C. macrophylla* plant.

### Mating experiments.

Four populations comprised of the following combinations were produced: females (Californian uninfected) × males (Californian uninfected), females (Hawaiian infected) × males (Californian uninfected), females (Californian uninfected) × males (Hawaiian infected), and females (Hawaiian infected) × males (Hawaiian infected). Newly emerged female adults from DcRV-infected or uninfected colonies were separated from males upon emergence and held for 10 days on clean *C. macrophylla* plants. Twenty of these females and 20 male adults from the DcRV-infected or uninfected colonies were placed together in a cage for 7 days to create each mating combination population. The parental insects were removed and the plants held for egg eclosion, nymphal development, and adult emergence. The total RNAs of newly emerged F_1_ adults were individually extracted using TRIzol reagent (Thermo Fisher Scientific). Three independent biological replicates were conducted and analyzed.

### RT-qPCR assays.

First-strand cDNA was synthesized using gene-specific primers (data not shown) and SuperScript II reverse transcriptase (Thermo Fisher Scientific). RT-qPCR assays were performed using Universal SYBR green supermix (Bio-Rad) in the CFX96 touch system (Bio-Rad). The mRNA of the *actin* gene of *D. citri* was used as the control for each relative quantification PCR assay. Quantitative analyses for the relative levels of gene accumulation were analyzed according to the 2^−ΔΔ^*^CT^* method.

For the quantification of DcRV in insects, the concentration of the plasmid DNA including the DcRV P8 gene was determined using a NanoDrop 1000 (Thermo Fisher Scientific). The copy number of the plasmid including the DcRV P8 gene was calculated using the following formula: (amount × 6.022 × 10^23^)/(length × 1 × 10^9^ × 650). A 10-fold dilution series was prepared in RNase-free water to establish a standard curve. The threshold cycle value was mapped to the standard curve of DcRV P8, and an equation was generated (Data Set S1). The viral genome copy as the log of the copy number per microgram of insect RNA was based on the equations.

### Recombinant baculovirus expression of DcRV P10.

The coding region of the open reading frame for DcRV P10 was ampliﬁed by RT-PCR with forward primer and reverse primer fused with the sequence of the His tag. The puriﬁed PCR product was cloned into the Gateway vector pDEST8 (Thermo Fisher Scientific) to generate a recombinant baculovirus vector containing P10. A recombinant bacmid was generated by introducing the recombinant baculovirus vector into Escherichia coli DH10Bac (Thermo Fisher Scientific). Sf9 cells were inoculated with the puriﬁed recombinant bacmid in the presence of Cellfectin II (Thermo Fisher Scientific) according to the manufacturer’s instructions. After a high-titer baculoviral stock was generated, Sf9 cells growing on coverslips were infected with recombinant bacmids. At 48 hpi, Sf9 cells were ﬁxed, permeabilized, and immunolabeled with fluorescein isothiocyanate-conjugated His-6×His tag antibody (Thermo Fisher Scientific). Cells inoculated with empty baculovirus vector served as a mock-infected control. The cells were observed with a Leica TCS SPE laser confocal microscope.

### Effect of synthesized dsRNAs on expression of target genes in female ovaries and DcRV accumulation in *D. citri* eggs.

To synthesize dsRNAs *in vitro*, the forward and reverse primers with a T7 RNA polymerase promoter sequence at the 5′ termini (data not shown) were used in PCR to amplify regions of ∼500 to 900 bp of the target genes. The T7 RiboMAX express RNAi system (Promega) was used to transcribe PCR products into dsRNAs. The integrity and quantity of puriﬁed dsRNAs were examined by agarose gel electrophoresis and UV spectroscopy, respectively.

Uninfected newly emerged Californian female *D. citri* adults were microinjected with a mixture of dsRNAs (0.2 μg/μl) and the 20% (wt/vol) crude extract of DcRV. At 2 dpi, these treated females were individually mated with uninfected California *D. citri* males on clean flush tips in separate cages. The plant material was replaced every 3 days to facilitate egg collection. To exclude the viral source in the hemolymph, ovaries of 20 females were dissected every 3 days for RNA isolation. RNA from the Direct-zol RNA kit (Zymo Research) was used to purify RNA from ovaries, and TRIzol reagent (Thermo Fisher Scientific) was used to purify total RNA from the eggs. RT-qPCR detection was conducted for relative gene accumulation of P10, P8, and Vg. The total proteins of 30 ovaries were extracted at 7 dpi for the Western blot analysis. More than 3 independent biological replicates were conducted and analyzed.

### Sperm extraction and incubation *in vitro*.

The testes and seminal vesicles were dissected individually from 20 DcRV-infected Hawaiian *D. citri* male adults in 0.1 M PBS (pH 7.2), and then the supernatant was collected by centrifugation at low speed. The sperm suspension drops were placed on Polysine adhesion slides (Thermo Fisher Scientific) and dried at room temperature. Immunofluorescence microscopy then was conducted.

For sperm incubation *in vitro*, the dissected testes and seminal vesicles of uninfected Californian *D. citri* male adults were incubated with 20% (wt/vol) DcRV crude extract from DcRV-infected Californian *D. citri* females at room temperature for 30 min. The sperm suspension drops then were placed on Polysine adhesion slides and processed for immunofluorescence microscopy.

### Effect of synthesized dsP10 on P10 gene accumulation in *D. citri* males and DcRV infection rate in progeny.

Newly emerged uninfected California *D. citri* female adults were held separately from males for 10 days. Uninfected California *D. citri* male adults were microinjected with a mixture of dsRNAs (0.2 μg/μl) and the 20% (wt/vol) DcRV crude extract. At 5 dpi, these treated males were placed with virgin uninfected California *D. citri* females on clean plants for 7 days. The testes of 20 males were dissected every 3 days for RNA isolation to exclude the viral source in the hemolymph. The total proteins of dissected testes derived from 40 males were extracted at 7 dpi for the Western blot analysis.

At 13 dpi, all parental *D. citri* insects were removed from the cages and the eggs and nymphs allowed to eclose and develop to the adult stage. The total RNAs of newly emerged F_1_ adults were individually extracted using TRIzol reagent (Thermo Fisher Scientific). Three independent biological replicates were conducted and analyzed.

### Statistical analyses.

All data for relative accumulation of genes were analyzed with two-tailed *t* test in GraphPad Prism 7. The data for percentage of vitellogenic ovary in dsRNA-treated females were analyzed with one-tailed *t* test in GraphPad Prism 7. The data were back-transformed after analysis for presentation in the text and figures.

## Supplementary Material

Supplemental file 1

## References

[1] Gottwald TR 2010 Current epidemiological understanding of citrus Huanglongbing. Annu Rev Phytopathol 48:119–139. doi:10.1146/annurev-phyto-073009-114418. 20415578

[2] Marutani-Hert M, Hunter WB, Katsar CS, Sinisterra XH, Hall DG, Powell CA 2009 Reovirus-like sequences isolated from adult Asian citrus psyllid, (Hemiptera: Psyllidae: Diaphorina Citri). Fla Entomol 92:314–320. doi:10.1653/024.092.0216.

[3] Nouri S, Salem N, Nigg JC, Falk BW 2016 Diverse array of new viral sequences identified in worldwide populations of the Asian citrus psyllid (Diaphorina citri) using viral metagenomics. J Virol 90:2434–2445. doi:10.1128/JVI.02793-15. PMC4810699 26676774

[4] Ter Horst AM, Nigg JC, Dekker FM, Falk BW 2018 Endogenous viral elements are widespread in arthropod genomes and commonly give rise to piRNAs. J Virol 93:e02124-18. doi:10.1128/JVI.02124-18. PMC6401445 30567990

[5] Attoui H, Mertens PPC, Becnel J, Belaganahalli S, Bergoin M, Brussaard CP, Chappell JD, Ciarlet M, del Vas M, Dermody TS, Dormitzer PR, Duncan R, Fcang Q, Graham R, Guglielmi KM, Harding RM, Hillman B, Makkay A, Marzachì C, Matthijnssens J, Milne RG, Mohd Jaafar F, Mori H, Noordeloos AA, Omura T, Patton JT, Rao S, Maan M, Stoltz D, Suzuki N, Upadhyaya NM, Wei C, Zhou H 2012 Family reoviridae, p 546–639. *In* King AMQ, Adams MJ, Carstens EB, Lefkowitz EJ (ed), Virus taxonomy: ninth report of the international committee for the taxonomy of viruses. Academic Press, New York, NY.

[6] Nakashima N, Koizumi M, Watanabe H, Noda H 1996 Complete nucleotide sequence of the Nilaparvata lugens reovirus: a putative member of the genus Fijivirus. J Gen Virol 77:139–146. doi:10.1099/0022-1317-77-1-139. 8558122

[7] Nakashima N, Noda H 1995 Nonpathogenic Nilaparvata lugens reovirus is transmitted to the brown planthopper through rice plant. Virology 207:303–307. doi:10.1006/viro.1995.1082. 7871743

[8] Noda H, Ishikawa K, Hibino H, Omura T 1991 A reovirus in the brown planthopper, Nilaparvata lugens. J Gen Virol 72:2425–2430. doi:10.1099/0022-1317-72-10-2425. 1919524

[9] Falk BW, Kim KS, Tsai JH 1988 Electron microscopic and physicochemical analysis of a reo-like virus of the planthopper Peregrinus maidis. Intervirology 29:195–206. doi:10.1159/000150046. 3182234

[10] Spear A, Sisterson MS, Stenger DC 2012 Reovirus genomes from plant-feeding insects represent a newly discovered lineage within the family Reoviridae. Virus Res 163:503–511. doi:10.1016/j.virusres.2011.11.015. 22142476

[11] Stenger DC, Sisterson MS, Krugner R, Backus EA, Hunter WB 2009 A new Phytoreovirus infecting the glassy-winged sharpshooter (Homalodisca vitripennis). Virology 386:469–477. doi:10.1016/j.virol.2009.01.037. 19239972

[12] Chen Q, Chen H, Mao Q, Liu Q, Shimizu T, Uehara-Ichiki T, Wu Z, Xie L, Omura T, Wei T 2012 Tubular structure induced by a plant virus facilitates viral spread in its vector insect. PLoS Pathog 8:e1003032. doi:10.1371/journal.ppat.1003032. 23166500 PMC3499585

[13] Jia DS, Mao QZ, Chen HY, Wang AM, Liu YY, Wang HT, Xie LH, Wei TY 2014 Virus-induced tubule: a vehicle for rapid spread of virions through basal lamina from midgut epithelium in the insect vector. J Virol 88:10488–10500. doi:10.1128/JVI.01261-14. 24965461 PMC4178856

[14] Wei TY, Li Y 2016 Rice reoviruses in insect vectors. Annu Rev Phytopathol 54:99–120. doi:10.1146/annurev-phyto-080615-095900. 27296147

[15] Cserzö M, Wallin E, Simon I, von Heijne G, Elofsson A 1997 Prediction of transmembrane alpha-helices in prokaryotic membrane proteins: the dense alignment surface method. Protein Eng 10:673–676. doi:10.1093/protein/10.6.673. 9278280

[16] Dossi FCA, Consoli FL 2014 Gross morphology and ultrastructure of the female reproductive system of Diaphorina citri (Hemiptera: Liviidae). Zoologia 31:162–169. doi:10.1590/S1984-46702014000200007.

[17] Kot M, Buning J, Jankowska W, Drohojowska J, Szklarzewicz T 2016 Development of ovary structures in the last larval and adult stages of psyllids (Insecta, Hemiptera, Sternorrhyncha: Psylloidea). Arthropod Struct Dev 45:389–398. doi:10.1016/j.asd.2016.04.004. 27140505

[18] Tufail M, Nagaba Y, Elgendy AM, Takeda M 2014 Regulation of vitellogenin genes in insects. Entomol Sci 17:269–282. doi:10.1111/ens.12086.

[19] Barcellos MS, Cossolin JFS, Dias G, Lino-Neto J 2017 Sperm morphology of the leafhopper Diaphorina citri Kuwayama (Hemiptera: Sternorrhyncha: Psylloidea: Liviidae). Micron 99:49–55. doi:10.1016/j.micron.2017.03.017. 28431332

[20] Noda H, Nakashima N 1995 Non-pathogenic reoviruses of leafhoppers and planthoppers. Semin Virol 6:109–116. doi:10.1006/smvy.1995.0014.

[21] Longdon B, Jiggins FM 2012 Vertically transmitted viral endosymbionts of insects: do sigma viruses walk alone? Proc R Soc B Biol Sci 279:3889–3898. doi:10.1098/rspb.2012.1208. PMC3427579 22859592

[22] Brun G, Plus N 1980 The viruses of Drosophila. Academic Press, New York, NY.

[23] Longdon B, Wilfert L, Obbard DJ, Jiggins FM 2011 Rhabdoviruses in two species of Drosophila: vertical transmission and a recent sweep. Genetics 188:U141–U227. doi:10.1534/genetics.111.127696. PMC3120147 21339477

[24] Lopez-Ferber M, Ferreiro Rios A, Kuhl G, Comendador MA, Louis C 1997 Infection of the gonads of the SimES strain of Drosophila simulans by the hereditary reovirus DSV. J Invertebr Pathol 70:143–149. doi:10.1006/jipa.1997.4683. 9281403

[25] Juchault P, Louis C, Martin G, Noulin G 1991 Masculinization of female isopods (Crustacea) correlated with non-Mendelian inheritance of cytoplasmic viruses. Proc Natl Acad Sci U S A 88:10460–10464. doi:10.1073/pnas.88.23.10460. 11607243 PMC52948

[26] Rosen L 1988 Further observations on the mechanism of vertical transmission of flaviviruses by Aedes mosquitos. Am J Trop Med Hyg 39:123–126. doi:10.4269/ajtmh.1988.39.123. 2840833

[27] Saiyasombat R, Bolling BG, Brault AC, Bartholomay LC, Blitvich BJ 2011 Evidence of efficient transovarial transmission of Culex flavivirus by Culex pipiens (Diptera: Culicidae). J Med Entomol 48:1031–1038. doi:10.1603/ME11043. 21936322

[28] Tesh RB 1984 Transovarial transmission of arboviruses in their invertebrate vectors. Praeger Publishers, Santa Barbara, CA.

[29] Goertz D, Solter LF, Linde A 2007 Horizontal and vertical transmission of a Nosema sp. (Microsporidia) from Lymantria dispar (L.) (Lepidoptera: Lymantriidae). J Invertebr Pathol 95:9–16. doi:10.1016/j.jip.2006.11.003. 17250850

[30] Prabhu S, Mahalingam CA 2017 Effect of sunlight and UV light against DpNPV (nuclear polyhedrosis virus) formulation on larval mortality of mulberry leaf Webber, Diaphania pulverulentalis Hampson. Int J Curr Microbiol Appl Sci 6:1897–1905.

[31] Wayne ML, Blohm GM, Brooks ME, Regan KL, Brown BY, Barfield M, Holt RD, Bolker BM 2011 The prevalence and persistence of sigma virus, a biparentally transmitted parasite of Drosophila melanogaster. Evol Ecol Res 13:323–345. 28217032 PMC5313041

[32] Chen Q, Wei T 2016 Viral receptors of the gut: insect-borne propagative plant viruses of agricultural importance. Curr Opin Insect Sci 16:9–13. doi:10.1016/j.cois.2016.04.014. 27720057

[33] Huo Y, Liu W, Zhang F, Chen X, Li L, Liu Q, Zhou Y, Wei T, Fang R, Wang X 2014 Transovarial transmission of a plant virus is mediated by vitellogenin of its insect vector. PLoS Pathog 10:e1003949. doi:10.1371/journal.ppat.1003949. 24603905 PMC3946389

[34] Jia DS, Mao QZ, Chen Y, Liu YY, Chen Q, Wu W, Zhang XF, Chen HY, Li Y, Wei TY 2017 Insect symbiotic bacteria harbour viral pathogens for transovarial transmission. Nat Microbiol 2:17025. doi:10.1038/nmicrobiol.2017.25. 28263320

[35] Wei J, He YZ, Guo Q, Guo T, Liu YQ, Zhou XP, Liu SS, Wang XW 2017 Vector development and vitellogenin determine the transovarial transmission of begomoviruses. Proc Natl Acad Sci U S A 114:6746–6751. doi:10.1073/pnas.1701720114. 28607073 PMC5495249

[36] Liao ZF, Mao QZ, Li JJ, Lu CC, Wu W, Chen HY, Chen Q, Jia DS, Wei TY 2017 Virus-induced tubules: a vehicle for spread of virions into ovary oocyte cells of an insect vector. Front Microbiol 8:475. doi:10.3389/fmicb.2017.00475. 28382031 PMC5360704

[37] Cui X, Yaghmaiean H, Wu G, Wu X, Chen X, Thorn G, Wang A 2017 The C-terminal region of the Turnip mosaic virus P3 protein is essential for viral infection via targeting P3 to the viral replication complex. Virology 510:147–155. doi:10.1016/j.virol.2017.07.016. 28735115

[38] Chapman RF 1969 The insects structure and function, 4th ed American Elsevier Publishing Company, New York, NY.

[39] Hagedorn HH, Kunkel JG 1979 Vitellogenin and vitellin in insects. Annu Rev Entomol 24:475–505. doi:10.1146/annurev.en.24.010179.002355.

[40] Longdon B, Jiggins FM 2010 Paternally transmitted parasites. Curr Biol 20:R695–R696. doi:10.1016/j.cub.2010.06.026. 20833306

[41] Teninges D 1968 Demonstration of sigma viruses in the cells of the stabilized male germinal line of Drosophila. Arch Gesamte Virusforsch 23:378–387. doi:10.1007/BF01242133. 5680979

[42] Klowden MJ 2009 Spermatheca, 2nd ed Academic Press, New York, NY.

[43] Mansuy JM, Suberbielle E, Chapuy-Regaud S, Mengelle C, Bujan L, Marchou B, Delobel P, Gonzalez-Dunia D, Malnou CE, Izopet J, Martin-Blondel G 2016 Zika virus in semen and spermatozoa. Lancet Infect Dis 16:1106–1107. doi:10.1016/S1473-3099(16)30336-X. 27676340

[44] Mao Q, Wu W, Liao Z, Li J, Jia D, Zhang X, Chen Q, Chen H, Wei J, Wei T 2019 Viral pathogens hitchhike with insect sperm for paternal transmission. Nat Commun 10:955. doi:10.1038/s41467-019-08860-4. 30814506 PMC6393494

[45] L’Heritier PH 1970 Drosophila viruses and their role as evolutionary factors. Evol Biol 4:185–209.

[46] Chen H, Zheng L, Jia D, Zhang P, Chen Q, Liu Q, Wei T 2013 Rice gall dwarf virus exploits tubules to facilitate viral spread among cultured insect vector cells derived from leafhopper Recilia dorsalis. Front Microbiol 4:206. doi:10.3389/fmicb.2013.00206. 23888157 PMC3719018

[47] Wei TY, Kikuchi A, Moriyasu Y, Suzuki N, Shimizu T, Hagiwara K, Chen HY, Takahashi M, Ichiki-Uehara T, Omura T 2006 The spread of Rice dwarf virus among cells of its insect vector exploits virus-induced tubular structures. J Virol 80:8593–8602. doi:10.1128/JVI.00537-06. 16912308 PMC1563882

